# A Comparison of Divergent Thinking Abilities Between Healthy Elderly Subjects and MCI Patients: Preliminary Findings and Implications

**DOI:** 10.3389/fpsyg.2020.00738

**Published:** 2020-04-30

**Authors:** Giulia Fusi, Elena Ferrari, Marina Zanetti, Maura Crepaldi, Carol Bersanini, Anna Paladino, Laura Colautti, Luca Rozzini, Alessandro Antonietti, Maria Luisa Rusconi

**Affiliations:** ^1^Department of Human and Social Sciences, University of Bergamo, Bergamo, Italy; ^2^Neurology Unit, Department of Clinical and Experimental Sciences, University of Brescia, Brescia, Italy; ^3^Department of Psychology, Catholic University of the Sacred Heart, Milan, Italy

**Keywords:** mild cognitive impairment, divergent thinking, creativity, aging, dementia, cognitive reserve

## Abstract

**Objective:**

Divergent thinking (DT) has attracted research interest because of its potential role in early diagnosis and rehabilitation programs for patients affected by neurodegenerative diseases. Recently, DT has received even more attention because of its proven relationship with cognitive reserve (CR) and the possibility of a standardized assessment. However, few studies have investigated this ability in dementia patients, and even less is known about patients affected by Mild Cognitive Impairment (MCI). Thus, this study aims to investigate DT abilities in MCI patients.

**Methods:**

A total of 25 MCI patients and 25 healthy controls subjects (HC; from a random selection of 50) matched for age, gender, and educational level were enrolled. General cognitive functioning was measured by the Montreal Cognitive Assessment (MoCA), while the Abbreviated Torrance Test for Adults (ATTA) was selected to measure DT.

**Results:**

MANOVA analysis did not reveal any significant differences in DT abilities between MCI patients and HC except for the figural indicator score. A logistic hierarchical regression analysis revealed that the figural indicator score added an 8% of accuracy in the prediction of the group variable over the general cognition measure (MoCA).

**Conclusion:**

MCI patients seem to perform significantly worse than HC only in the figural DT score and this evidence has significant practical implications. First, that figural DT seemed to decrease even earlier than verbal DT and could therefore be taken into account for early diagnosis of MCI patients. On the contrary, the sparing of all the other DT skills (such as verbal DT skills, fluency, flexibility, originality, and elaboration) may suggest that, given its relationship with CR, verbal DT could instead be considered a possible target for prevention or early cognitive stimulation interventions.

## Introduction

The connection between mental health and creativity has traditionally been studied in terms of qualitative examination of novel artistic productions of patients affected by neurodegenerative pathologies or through the evaluation of changes in the artistic style of artists who suffered from these diseases (see Palmiero et al., 2012; Miller and Miller, 2013; Acosta, 2014; de Souza et al., 2014; Abraham, 2019 for a review). Going beyond an initial impetus to find a direct link between neurodegenerative diseases and creativity, it was recently observed that the “*de novo*” productions in this type of patient are rare and that almost none of them could be considered extraordinary (Abraham, 2019). Consequently, researchers have begun to consider the artistic expression exhibited by these patients only as a way that reflects their need of some form of communication, which does not necessarily end up with the production of a creative product (Zaidel, 2014). Moreover, several authors consider it as a general “drive” to produce (Canesi et al., 2012) or as a “pseudo-creative production” triggered by cognitive or behavioral characteristics such as perseveration or disinhibition specifically related to the disease (de Souza et al., 2010, 2014).

It has also been pointed out that creativity is a complex, composite, and multidimensional construct, and it has been argued that it cannot be addressed only by qualitative observations, highlighting the need for the use of quantitative standardized methods (Torrance, 1988; Dietrich, 2004; Palmiero et al., 2012; Abraham, 2016; Barbot et al., 2019). Consequently, the construct of Divergent Thinking (DT), first proposed by Guilford in 1956, has been considered in a great number of experimental studies. DT was defined as the ability to generate many original, different, and elaborate responses to an open-ended question (Guilford, 1956, 1957, 1967). Nowadays, researchers consider DT as an indicator of creative potential (Runco and Acar, 2012; Acar and Runco, 2019): thus, not a synonym for creativity but an affordable predictor of future creative achievement (Kim, 2008). Furthermore, DT it is widely employed in the experimental field because it is believed to elicit the cognitive processes that lead to creative idea generation (Barbot et al., 2019; Benedek and Fink, 2019) and because it can be easily measured by psychometrical tools (Torrance, 1988; Acar and Runco, 2019; Barbot et al., 2019).

However, it should be noted that most of the contributions have experimentally analyzed DT abilities in young adults; fewer experimental studies have been focused on the elderly. In this population, the results are complex and sometimes inconsistent due to the great differences between experimental studies (Fusi et al., under review). Indeed, it seemed that early studies agreed on the evidence of a curvilinear decline in DT abilities during the life-span, with a peak around the 1950s and then a decline during late adulthood (McCrae et al., 1987), whereas some recent studies, which have considered more intervening variables (i.e., working memory, speed of elaboration, and so on) and a differential role of the aging processes on verbal DT versus figural DT, seem to show that DT skills are at least partially preserved during adulthood and the last decades of life (Palmiero et al., 2017), even if figural abilities appeared the most affected (Palmiero et al., 2014).

Divergent thinking has also recently received renewed attention because of its potential for early diagnosis (Hart and Wade, 2006) and rehabilitation programs in patients affected by different neurological diseases, particularly by dementia (Palmiero et al., 2012). Moreover, recent evidence has highlighted a significant and positive correlation between DT abilities and the construct of Cognitive Reserve (CR; Meléndez et al., 2016; Palmiero et al., 2016; Colombo et al., 2018), which is a pivotal concept in the field of aging. CR is indeed considered a protective factor against cognitive decline and refers to a functional benefit (rather than structural, i.e., brain reserve) associated with different life experiences, such as educational level, occupation, and cognitively stimulating leisure activities, that seem to provide protection against the effects of brain damage or pathology; thus, people with higher CR are believed to cope better with potential brain damage by recruiting compensatory processes (Stern, 2002, 2009, 2014). As a result of this proven relationship, DT has been proposed as a possible target for cognitive stimulation interventions. Exercising divergent and creative thinking has been taken into consideration as a way to promote mental health and active aging by fostering creative cognition in daily life (Cropley, 1990), thereby reducing the risk of dementia (Palmiero et al., 2016), and also to try to slow down cognitive decline during neurodegenerative diseases (Palmiero et al., 2012; Ruggiero et al., 2019).

In spite of this significant evidence, few studies have evaluated DT abilities through standardized tests in patients affected by different types of dementia (Bigler et al., 1988; Bigler, 1995; Rankin et al., 2007; de Souza et al., 2010; Ruggiero et al., 2019) and studies focusing on DT in the early phases of the disease are even more scarce (Hart and Wade, 2006). Almost all of the studies highlighted a decline in DT abilities in this type of patient, even in the early stages (Hart and Wade, 2006). In light of this, early assessment and intervention in patients at risk of dementia could be considered crucial. However, to our knowledge, no research has studied DT abilities in patients affected by MCI, which is generally considered to be a transitional stage between normal and pathological aging (see Petersen, 2016 for a review) or as a prodromal stage of the onset of Alzheimer’s disease (AD, Petersen et al., 2001). This assessment might be particularly important when considering data on the prevalence and conversion rate to dementia in MCI patients; indeed, even if these data vary greatly according to the different definitional applied criteria (Bischkopf et al., 2002), epidemiological studies have estimated that the prevalence of MCI in the elderly population ranges from 3 to 19% (Gauthier et al., 2006; Colautti et al., 2018) and that 11 to 49% of people with MCI progressed to dementia (mainly AD) within 2 years (Gauthier et al., 2006; Bondi et al., 2014). Therefore, it is considered a priority to find early cognitive markers for the diagnosis of MCI (see, for example, Arnáiz and Almkvist, 2003) and also to develop training and cognitive stimulation programs that might help these patients to compensate for their cognitive difficulties. Cognitive stimulation training could, in fact, offer to these patients some protection against cognitive decline by stimulating pre-existing neural reserves or recruiting neural circuitry as compensatory scaffolding (Reuter-Lorenz and Park, 2014; Sherman et al., 2017), promoting brain plasticity (Li et al., 2011), and could thereby reduce the risk or delay the progression of dementia (Anderson, 2019). Consequently, the aim of this study was to examine DT abilities in a sample of patients diagnosed with MCI.

## Materials and Methods

### Participants

The study involved a total of 75 participants: 25 MCI patients (MCI) and 50 Healthy Control subjects (HC). However, due to the lack of proportionality of the two samples, a random selection of 25 subjects from the HC group was performed. Their socio-demographical data are summarized in Table 1. No differences between the groups emerged both for demographic factors (i.e., age, sex, and educational level) or psychological variables such as apathy, anxiety, or depression, respectively measured by the Apathy Evaluation Scale (AES, Marin et al., 1991), the State-Trait Anxiety Inventory (STAI, Spielberger et al., 1983; Pedrabissi and Santinello, 1989), and the Beck Depression Inventory (BDI, Beck et al., 1961).

**TABLE 1:** Sample demographic data.

Variables	MCI (*N* = 25)	HC (*N* = 25)	*t*(48), U	p
Age	75.32 ± 5.47	74.16 ± 5.35	−0.758	0.45
Educational level	8.40 ± 4.34	7.44 ± 4.50	−0.768	0.45
Gender	13 women; 12 men	19 women; 6 men	237.50	0.08
AES	34.12 ±	31.84 ± 5.84	−1.162	0.25
STAI	35.88 ± 10.23	39.16 ± 11.67	1.057	0.30
BDI	6.36 ± 6.55	9.20 ± 5.46	1.665	0.10

*STAI, State-Trait Anxiety Index; AES, Apathy Evaluation Scale; BDI, Beck Depression Inventory.*

Mild Cognitive Impairment patients were recruited at the Neuropsychology Service of the ASST Spedali Civili in Brescia (Italy), while HC subjects were enrolled from a pool of older adult volunteers. The MCI group was composed by subjects diagnosed as affected by Mild Cognitive Impairment according to Petersen’s criteria (Petersen et al., 1999; Petersen, 2004), which include subjective or proxy cognitive complaint and objective impairment in memory and/or at least one other cognitive domain; furthermore, patients must have a relatively intact capability to perform basic and instrumental daily life activities independently. All patients underwent an extensive neuropsychological assessment in order to verify these conditions and were screened for inclusion criteria, which included: age >50 years old, Mini-Mental State Examination >22 (MMSE; Folstein et al., 1975; Measso et al., 1993) and no sign of vascular lesions and of neurological or psychiatric diseases. All tests were administered to the whole sample individually and in a single session that lasted approximately 1 h; the evaluation was performed in a quiet setting. An informed consent form was signed by all of the participants. The research protocol and procedure were approved by the Hospital Ethical Committee and were conducted according to the Declaration of Helsinki.

### Materials

#### Cognitive Measures

##### Montreal Cognitive Assessment (MoCA)

The MoCA (Santangelo et al., 2015) test was designed to evaluate general cognition; this test is considered a sensitive tool for the detection of MCI (Boccardi et al., 2020). It is composed of 12 sub-tasks that evaluate different cognitive functions such as attention, executive functions, memory, language, abstraction, calculation, and orientation and visuo-constructional abilities (max score = 30).

#### Divergent Thinking Measure

##### Abbreviated Torrance Test for Adults (ATTA)

The ATTA test (Goff, 2002) was selected to evaluate divergent thinking abilities and therefore the creative potential of the subjects. ATTA is the abbreviated form of the famous Torrance Test of Creative Thinking (Torrance, 1988), which is the instrument that is most widely used by researchers to assess creativity and its reliability and predictive validity have been already proved (see Kim, 2006 for a review). ATTA was chosen due to the time limitation in clinical practice; it requires a short time for administration and had already been used in the Italian clinical context by Canesi et al. (2016, 2017) and Colautti et al. (2018). This test consists of three different tasks: the first is a verbal task, also called “consequences,” in which subjects are asked to list all the problems that could arise following an implausible situation assumed to be true such as “if you could fly or walk on air without being on an airplane or in a similar vehicle.” The second and third tasks evaluate figural DT abilities: the participants are asked to make a drawing starting from two incomplete figures and to give a title to their production; in the third task, nine equal triangles are given to the participants, who are asked to use them to create different figures, objects, or images. For this task, they are also asked to give a title to every drawing. The instruction to “be creative” is given before the start of every administration, and the time given to perform each task is 3 min.

The scores and indices of the sub-scales of fluency, originality, processing have been calculated according to the manual’s norms. Moreover, the evaluation of creativity indicators for verbal (richness and colorfulness of imagery, emotions/feelings, future orientation, humor: conceptual incongruity, and provocative questions) and for figural (openness: resistance to premature closure, unusual visualization, movement or sound, richness of imagery, abstractness of titles, articulateness in telling a story, combination of two/more figures, internal visual perspective, expressions of feelings and emotions, and fantasy) tasks were computed (verbal indicators; figural indicators). The sum of the creative indexes and creative indicators constitute the total score, the Divergent Thinking Total Score (DTTS).

### Data Analysis

Data analyses were performed using SPSS software (version 24.0.0). Normality was checked for all the variables with the Shapiro-Wilk test and the examination of the skewness and asymmetry indexes were performed. Furthermore, a check for outliers was run. Almost all of the variables met the assumptions of normality; transformations (square root) were performed when necessary, and parametric tests were then used for all the analyses. To ensure that the two groups (HC vs. MCI) did not differ with regards to important demographic data, two independent *t*-tests were performed and no significant differences were found for age and years of education; a Welch’s *t*-test was used to compare gender, which was found to be non-significant (see Table 1). To assess for significant differences between the two groups in cognitive and psychological variables, different independent *t*-tests were performed. The homogeneity of variances assumption was checked using Levene’s Test. Considering the high correlations (greater than 0.30) between the different DT indexes, a multivariate analysis of variance (MANOVA) was chosen to test between-group differences in these variables. Finally, a hierarchical logistic regression was run to evaluate the possible predictive role of cognitive and DT measures to discriminate between the two groups.

## Results

As could be predicted from the previous literature, the MOCA scores differed significantly, *t*(48) = 3.762, *p* < 0.001 between MCI patients and HC groups, with MCI patients performing worse than HC (see Table 2).

**TABLE 2:** Descriptive statistics and between-group differences for general cognition and divergent thinking measures.

Measure	MCI	HC	t (76)	F	p
**General cognition**					
MoCA	18.88 ± 3.54	22.12 ± 3.60	3.210		0.01
**Divergent thinking**					
ATTA_DTTS	51.52 ± 5.06	54.28 ± 7.62			
ATTA_Fluency	6.72 ± 3.01	8.80 ± 4.44		3.756	0.06
ATTA_ Flexibility	1.36 ± 1.32	2.08 ± 2.16		2.025	0.16
ATTA_Originality	3.24 ± 2.13	3.76 ± 2.47		0.636	0.43
ATTA_Elaboration	0.84 ± 1.07	1.04 ± 1.57		0.278	0.60
ATTA_Verbal_indicator	0.60 ± 0.65	0.76 ± 0.83		0.578	0.45
ATTA_Figural_indicator	1.32 ± 1.22	2.08 ± 1.04		5.655	0.02

**FIGURE 1:**
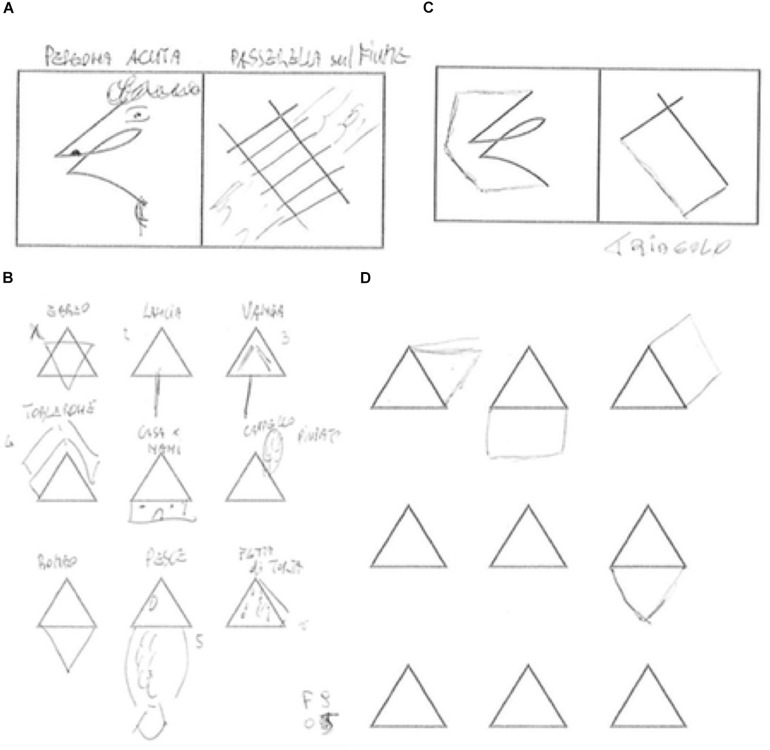
Figural indicator examples. **(A)** Task 2 and **(B)** Task 3 from an HC participant, score = 4:2 points for resistance to premature closure, 1 point for unusual perspective, and 1 point for abstractness of title, “sharp person.” **(C)** Task 2 and **(D)** Task 3: MCI patient, score = 0.

A MANOVA was performed with fluency, flexibility, originality, and elaboration scores and verbal and figural indicator scores of the ATTA test as dependent variables and “group” treated as the between factor variable. The analysis returned a non-significant effect of group, Wilks’ Lambda = 0.830, *F*(6,43) = 1.472, *p* > 0.05. Univariate ANOVA revealed that only the figural indicator score was significantly affected by group, *F*(1,48) = 5.655, *p* < 0.05, with MCI patients performing significantly worse (*M* = 1.32, *SD* = 1.22) than HC (*M* = 2.08, *SD* = 1.04). A comparison between drawings from one MCI patient and one matched HC subject is presented in Figure 1. The fluency, *F*(1,48) = 3.756, *p* > 0.05, originality, *F*(1,48) = 0.636, *p* > 0.05, elaboration, *F*(1,48) = 0.278, *p* > 0.05, flexibility, *F*(1,48) = 2.025, *p* > 0.05, and verbal *F*(1,48) = 0.578, *p* > 0.05 indicators were not affected by the independent variable.

Considering this first exploratory result, a hierarchical logistic regression was run in order to check for the possible predictive value of Figural Indicator, over the general cognition variable (MoCA), as a predictor of the group variable. Table 3 reports the regression results.

**TABLE 3:** Hierarchical Logistic Regression analysis predicting the dependent variable “group.”

	95% CI for Odds ratio				
Variables	Lower CI	Exp (B)	Upper CI	Wald	Sign.
**Model 1**					0.002
Intercept		0.988		0.002	
MoCA	0.183	0.371	0.752	7.575	0.006
**Model 2**					0.001
Intercept		3.226		3.402	0.65
MoCA	0.160	0.344	0.739	7.489	0.006
Figural_Indicator	0.273	0.504	0.931	4.784	0.029

Model 1 tests the effects of MoCA as the only predictor of the dependent variable “group.” The estimated coefficients for Model 1 indicate that MoCA significantly predicted the dependent variable (Wald = 7.58, *p* < 0.05) and accounted for 68% of correctly classified cases. A second block was then added so that Model 2 tested the predictive effects of the Figural Indicator predictor on the dependent variable “group” over the effect of MoCA. The change in the amount of information explained with this second model is significant [χ^2^(1) = 15.06, *p* < 0.001]. The significance values of the Wald statistics of the two predictors indicate that both the MoCA (Wald = 7.49, *p* < 0.05) and Figural Indicator (Wald = 4.78, *p* < 05) significantly predict the dependent variable; furthermore the odds ratios of MoCA, (Exp(B) = 0.34, CI_0.95_ = [0.16,0.74]) and Figural Indicator (Exp(B) = 0.50, CI_0.95_ = [0.27,0.93]) indicate that if the value of MoCA or Figural Indicator goes up by 1 point, the odds of being part of the HC group increase. Together, the predictors accounted for 76% of correctly classified cases.

## Discussion

The present study aimed to preliminarily explore the possibility that divergent thinking skills are affected in MCI patients, considering all the indices and indicators (i.e., fluency, flexibility, originality, elaboration; verbal versus figural indicators). The between-groups comparison has highlighted that MCI patients performed worse only in the figural indicator score. This result is partially in line with previous studies that have already proved the impairment of visual DT in dementia of Alzheimer’s type (Bigler et al., 1988; Bigler, 1995) and have hence suggested the possibility that a design fluency test could be used as a sensitive measure of performance deficit in these types of patients. Similarly, Hart and Wade (2006) have assessed verbal DT abilities in the early phases of AD and fronto-temporal dementia (FTD). However, no one has evaluated DT abilities in prodromal phases like MCI until now. The fact that the figural indicator score is already impaired in these patients might represent another piece of evidence for the value of considering poor performance in complex figural DT tasks as an early sign (even earlier than verbal ones) of cognitive impairment. Indeed, our analysis showed that the predictive value of the figural indicator score added 8% to the accuracy of detection of MCI patients in addition to the MoCA test prediction, which is considered the most sensitive tool for the detection of slight cognitive impairment (Boccardi et al., 2020). This is particularly relevant for the field, because the discovery of the long preclinical phase of AD and the other types of dementia has led to an enhanced interest in establishing early diagnostic indices of dementia (Bischkopf et al., 2002). Thus, figural DT abilities might be considered by future research as an early cognitive marker; nevertheless, more experimental studies are needed in order to confirm this result and to confirm the predictive value of visual DT abilities.

Interestingly, if a worse performance emerged in the figural indicator score in MCI patients, all the other indexes (i.e., fluency, originality, flexibility, and elaboration) and the verbal indicator score seemed to be unaffected. These results are partially in line with the complex and sometimes inconsistent studies that have evaluated the impact of the aging processes on DT abilities (Fusi et al., under review). Some authors highlighted a major impact of normal aging on visual DT abilities compared to verbal ones (Palmiero et al., 2014) or at least an almost linear decline in visual abilities (Palmiero et al., 2017). These results also agree with previous studies concerning the aging brain, which have already shown how verbal abilities could remain intact across the lifespan (Kemper and Kemtes, 1999; Park et al., 2002) or at least not encounter a decline until late in life (Hedden and Gabrieli, 2004). Moreover, elderly people seemed to perform significantly better than young people in verbal tasks, whereas they performed worse in visual tasks (Passafiume et al., 2010); as a result, it seems that figural DT is more affected even in MCI patients. Additionally, this result also seems to be in line with the existing literature about the relationship between DT and CR, which has evidenced that verbal (Palmiero et al., 2016; Colombo et al., 2018) but not visual (Palmiero et al., 2016) DT predicts CR. Thus, the idea that CR can be generally related to verbal ability (Palmiero et al., 2016), has already been advanced; it is consequently possible to hypothesize that CR might have a protective role on verbal (but not figural) DT abilities during early and prodromal stages of the disease, allowing patients to perform in a way comparable to control subjects. Moreover, the fact that this type of patient could perform like healthy elderly subjects in all the other DT indexes, which are considered fundamental to the ability to produce divergent and creative responses, is certainly a significant result and might have important practical implications. It has already been highlighted that DT may be considered a proxy of CR, and that this, in turn opened up the possibility that cognitive stimulation, which aims at reducing the risk of dementia, might also rely on creative cognition (Palmiero et al., 2016). CR, indeed, is considered as a protective factor against cognitive decline that could always be heightened (Stern, 2002, 2009, 2014), even during the course of a neurodegenerative pathology (Liberati et al., 2012). Thereby, the sparing of verbal DT might be considered as a target for future research that tries to design cognitive training and stimulation programs. This also means that among the complex mental activities that are certainly involved in CR, activities that involve a divergent way of thinking and the generation of creative ideas could be a pivotal protective factor against the cognitive decline. Moreover, the potential beneficial effect of cognitive training to enhance cognitive and functional abilities for these types of patients has already been highlighted by several reviews and meta-analyses (see, for example, Jean et al., 2010; Li et al., 2011; Sherman et al., 2017). Thus, the stimulation of verbal DT abilities (and consequently of CR) or, more specifically, the enhancement of verbal proficiency and verbal abilities to think of different and creative original solutions might also help MCI patients to develop new, useful and flexible cognitive strategies (Palmiero et al., 2016) as well as to cope with the problems of their daily lives.

In conclusion, our preliminary results highlight a slight impairment in DT abilities in patients affected by MCI, but only in the figural indicator score. Thus, figural abilities seemed to be affected earlier than verbal ones in mild pathological aging. This result has two significant practical implications: first, figural DT might be considered for the early diagnosis of MCI patients and, secondly, the sparing of all the other DT abilities (i.e., verbal DT abilities, fluency, flexibility, originality, and elaboration) may suggest that, considering its relationship with CR, verbal DT could be considered as a possible and meaningful target for prevention or early cognitive stimulation programs.

## Limitations and Future Research Directions

Some limitations have to be reported: first of all, the sample size for each group was relatively small and could not be fully representative of the population. Secondly, a mixed MCI group was enrolled; further analysis considering the different subtypes of MCI (amnestic vs. non-amnestic, single domain vs. multiple domains; Petersen, 2016) might be important to clarify whether the reported results can be generalized to all types of MCI patients or if the different subtypes are linked to diverse impairment in DT abilities. Future research might consider to assess the main cognitive functions that are believed to be involved during DT tasks (i.e., attention, memory, executive functions, etc.) to determine the relationship between specific cognitive functions and DT abilities in the different subtypes of MCI patients. Finally, although our results showed that the figural indicator score is impaired in MCI patients, more experimental studies are needed to confirm this result, especially by using different measures that address DT figural abilities.

## Data Availability Statement

The raw data in the dataset supporting the conclusion of this manuscript and the cognitive and behavioral measure for MCI patients will be made available upon request.

## Ethics Statement

The studies involving human participants were reviewed and approved by Ethic Committee of the ASST Spedali Civili of Brescia. The patients/participants provided their written informed consent to participate in this study.

## Author Contributions

MR, LR, and AA were responsible for the study design. GF, EF, and MR wrote the manuscript. MZ was responsible for the patients’ assessment. GF, MC, and LC were responsible for the healthy controls’ assessment. EF and CB contributed to the assessment. AP critically revised the manuscript.

## Conflict of Interest

The authors declare that the research was conducted in the absence of any commercial or financial relationships that could be construed as a potential conflict of interest.
